# Effect of Apnea-Induced Hypoxia on Cardiovascular Adaptation and Circulating Biomarkers of Oxidative Stress in Elite Breath-Hold Divers

**DOI:** 10.3389/fphys.2021.726434

**Published:** 2021-09-09

**Authors:** Magdalena Solich-Talanda, Aleksandra Żebrowska, Rafał Mikołajczyk, Sabina Kostorz-Nosal, Dariusz Ziora, Dariusz Jastrzębski, Piotr Siermontowski

**Affiliations:** ^1^Department of Physiological and Medical Sciences, Academy of Physical Education, Katowice, Poland; ^2^Institute of Sport Sciences, Academy of Physical Education, Katowice, Poland; ^3^Department of Lung Diseases and Tuberculosis, Faculty of Medical Sciences in Zabrze, Medical University of Silesia, Zabrze, Poland; ^4^Department of Underwater Works Technology, Polish Naval Academy, Gdynia, Poland

**Keywords:** freediving, cardiac function analysis, oxidative stress, hypoxia, heat shock (stress) proteins

## Abstract

Given the previous evidence that breath-hold diving is a cause of physiological stress, this study aimed to determine whether a combination static and dynamic apnea would affect total oxidant status, nitric oxide, heat shock proteins and cardiovascular parameters in elite freedivers. Thirteen finalists of the World and European championships in swimming pool breath-hold diving participated in the study. Whole-body plethysmography and electrocardiography was performed to determine the cardiorespiratory variables at baseline and during the simulation static apnea. An assessment of the heart rate, blood oxygen saturation and biochemical variables was performed before and in response to a combination of a static followed by a dynamic apnea. Static and dynamic breath-holding had a significant effect on oxidative stress, as evidenced by an increase in the total oxidant status/capacity (*p* < 0.001). The post apnea concentrations of heat shock proteins 27 (HSP27) were significantly elevated (*p* < 0.03, but total antioxidant status (TAS), HSP90, HSP70, and nitric oxide (NO) changes were not significant. levels under the influence of the static and dynamic breath-hold protocol. A significant positive correlation between HSPs and TAS (*r* = 0.63; *p* < 0.05) as well as NO levels was associated with beneficial cardiovascular adaptation. An increase in serum HSP27 levels mediated in nitric oxide levels could explain its important role in improving cardiovascular functions in elite freedivers. Further studies are necessary to explain the exact mechanisms of breath holds training of cardiovascular adaptation responsible for maintaining adequate oxygen supply in elite divers.

## Introduction

Individual and team world freediving championships include disciplines, such as Static Apnea (STA) in which the diver holding his breath for as long as possible with his nose and mouth immersed, or Dynamic Apnea in which the diver travels underwater attempting to cover the greatest possible distance with or without fins (Elia et al., 2021). The trends in improving world records in this sport indicate specific training protocol including endurance training and breath-hold training with hypoxia exposition (Heusser et al., 2009; Cross et al., 2013). The diving reflex is a protective, multifaceted physiologic response whose aim is to preserve oxygen stores during times of water immersion. It is characterized by bradycardia, apnea, and increased peripheral vascular resistance which redistribute blood to the brain and hart while limiting oxygen consumption by non-essential muscle groups (Eichhorn et al., 2017; Vega, 2017). The reflex mechanism and physiological adaptation depend on various diving conditions (i.e., depth of diving, static or dynamic apnea, and water temperature). In well-trained freedivers, the ability to reduce oxygen saturation in the blood relatively slowly (Schagatay, 2011; Engan et al., 2013), the centralization of blood circulation, and bradycardia by stimulation of the trigeminal nerve (Buchholz et al., 2017) are important mechanisms to increase hypoxic tolerance. However, muscle contraction and higher energy demand during dynamic apnea decrease apnea tolerance.

The precise molecular changes responsible for cardiovascular adaptation in response to the independent and combined contribution of two factors, i.e., exercise and hypoxic exposure during static and dynamic apnea are still not well understood (Costalat et al., 2017; Bain et al., 2018; Elia et al., 2019; Cialoni et al., 2021b). It has been suggested that hypoxia and exercise alter molecular compounds of tissues, such as nitric oxide (NO) and endothelial relaxation factor (EDRF) levels. NO is known to play a crucial role in cytoprotection through its vasodilatation effect and its ability to modulate mitochondrial function. Exposure to hypoxia can stimulate the release of NO and increase inducible NO synthase (iNOS) gene expression, which has been suggested as the beneficial endothelial-dependent vasodilatation mechanisms in response to lowering oxygen availability (Lundberg et al., 2015). It has been evidenced that NO might play an important role in optimizing oxygen transport and restoration of arterial oxygen saturation after repeated breath-hold diving at a depth (Theunissen et al., 2013a; Cialoni et al., 2019; Mrakic-Sposta et al., 2019).

Some cardiovascular effects have been explained by the result of binding NO to the superoxide anion (O_2_^2+^) and reducing oxidative stress. Holding the breath during diving can cause adverse changes resulting from too long exposure to hypoxia and hypercapnia. These changes include the imbalance between the antioxidant capacity and the generation of reactive oxygen species (ROS; Cialoni et al., 2021a). Moreover, exposure to oxidative stress increases during the first breath when divers re-surface and the oxygen-deprived cells are flooded with oxygen. Tissue damage from ischemia and reperfusion, where there is an increased ROS production, might be partially inhibited by the higher release of NO and increase expression of heat shock proteins (HSPs; Inaguma et al., 1995; Djurhuus et al., 2010).

The induction of proteins from the HSP 27, HSP 70, and HSP 90 family in cardiomyocytes, endothelial cells and in the coronary vessels has a cytoprotective effect which is important in reducing the rate of stress factors-induced apoptosis (Snoeckx et al., 2001). Different functions of HSPs have been described to explain their physiological functions in response to hypoxia, including their role in the regulation in protein folding, oligomerization, translocation as well as anti-apoptotic properties (Lin et al., 2001; Arya et al., 2007).

Despite much-published data concerning the role of the above-mentioned cytoprotection in response to exercise training (Krüger et al., 2019), there is little information available on the effects of breath-hold training on oxidative stress, the antioxidant capacity of the blood, and the concentration of HSPs in breath-hold divers.

This study aimed to assess the relationship between the total antioxidant status (TAS), the NO levels, the concentration of HSPs and cardiovascular adaptation in response to a combination of a static followed by a dynamic apnea in elite freedivers.

## Materials and Methods

### Subjects

Thirteen elite freedivers (three women and ten men) mean age: 35.8 ± 5.7 years, body height 180.5 ± 8.8 cm, body mass 78.3 ± 17.0, BMI 23.8 ± 3.6) participated in the study. They were the members of the National Team and finalists of the World and European championships in swimming pool breath-hold diving (AIDA, 2021). The sample size reflect the target population of total members of Polish Freediving Association who have regularly competed if freediving competitions prior to the study. The sample size (*n* = 13) were calculated with confidence level 95% and the confidence interval 20% from the total population of 40 freedivers.

The training status of the subjects was 7.2 ± 2.0 years and the experience of freediving coincided with the career length in competition was 7.1 ± 2.0 years. The training protocol of freedivers comprised three components: (1) aerobic endurance training (stationary cycling, treadmill, and swimming training with intensity of individual 70–80%, (2) STA and different disciplines of dynamic apnea training, (3) strength training, and (4) hypoxic training with increase the time of hypoxia exposition from 20% in preparatory training period to 70% of maximal BHT freediving training in specific and pre- competitive phase of training.

They were the members of the National Team and finalists of the World and European championships in swimming pool breath-hold diving (AIDA, 2021). The mean best results in the three freediving competitions were: STA 6.25 ± 1.29 min (minimum 5.28 min; maximum, 9.35 min.), distance without fins (DNF) 148.92 ± 45.09 m (minimum, 88.0 m; maximum 244.0 m), and the distance with fins 190.92 ± 60.56 m (minimum, 106 m; maximum, 300 m). All participants had valid medical certificates qualifying them to practice freediving. Two weeks prior to the study, subjects were asked to consume the recommended mixed diet. The daily intakes for energy were 30–35 kcal/kg/day with the proportion of protein, lipids, and carbohydrates 20, 20, and 60%, respectively. The diet were formulated with food items commonly available. Three weeks before the study and during the study protocol none of the respondents consumed supplements that would additionally modify an endogenous antioxidant protection.

All subjects were instructed to abstain from exercise within 24 h before the biochemical measurements.

The participants’ age, height, body mass, body mass index (BMI), body composition, and training status are presented in Table 1. The body composition of all participants was evaluated using a model In Body220 analyzer (Biospace Inc., Seoul, Korea). At the baseline before the study protocol, the graded treadmill exercise test (HP/Cosmos-Pulsar, Germany) was performed to measure individual maximal oxygen uptake (VO_2max_) (Matalyzer 3B, Cortex, Germany).

**TABLE 1:** Characteristics of subjects.

**Variables**		**Age (year)**	**Height (cm)**	**Weight (kg)**	**BMI (kg/m^2^)**	**FAT (%)**	**FFM (kg)**	**TBW (kg)**	**BSA (m^2^)**	**VO_2max_ (ml/kg/min.)**	**Training status (year)**
*n* = 13	*X*	35.8	180.5	78.3	23.8	13.9	67.8	49.5	2.0	41.8	7.2
	SD	5.7	8.8	17.0	3.6	6.0	13.4	9.7	0.3	4.7	2.0

*BMI, body max index; FAT, fat mass; FFM, free fat mass; TBW, total body water; BSA, body surface area; VO_2*max*_, maximal oxygen uptake.*

Before entering the study, lung function and cardiovascular variables were assessed. Whole-body plethysmography was used to measure lung volumes and diffusing capacity for carbon monoxide (Elite Platinum, Med. Graphics 2010, United States) according to ATS/ERS guidelines (Miller et al., 2005; Table 2). Electrocardiography (System ECG RScribe5, MDS Cardio, United States) was performed to evaluate the selected variables of cardiac function at baseline and during the simulation STA test (Table 3). At baseline, arterial oxygen saturation (SaO_2_) (Konica Minolta PULSOX-300i, Japan) and systolic and diastolic blood pressure (SBP/DBP) (HEM-907 XL, Omron Corporation, Kyoto, Japan) were measured in all subjects.

**TABLE 2:** Lung function assessment.

**Variables**		**TLC (l)**	**TLC Pred (%)**	**RV (l)**	**RV Pred (%)**	**IC (l)**	**IC Pred (%)**	**ERV (l)**	**ERV Pred (%)**	**DLCO (ml/min/mmHg)**	**DLCO Pred (%)**
*n* = 13	*X*	8.3	118.7	1.8	96.8	4.6	134.3	1.9	128.4	39.6	115.0
	*SD*	1.5	1.5	0.4	22.9	1.2	19.1	0.7	50.2	10.5	20.3

*TLC, total lung capacity; RV, residua volume; IC, inspiratory capacity; ERV, residual expiratory volume; DLCO, diffusing capacity for carbon monoxide; Pred, predicted values.*

**TABLE 3:** Electrocardiographic cardiac function at baseline (REST) and in response to simulation static apnea test (STA).

**Variables**	**REST**		**STA**	
	***x***	**SD**	***x***	**SD**
HR (b/min)	66.0	12.0	62.0	22.0
PR (ms)	164.0	18.0	179.0	70.0
QRS (ms)	103.0	16.0	106.0	20.0
QT/QTC	1.1	0.1	1.0	0.1
Mean RR (ms)	924.0	167.0	1059.0	335.0
QTcB (ms)	415.0	31.0	407.0	60.0

*HR, heart rate; PR, interval represents the time between the onset of atrial depolarization and the onset of ventricular depolarization; QRS, ventricular depolarization; QTc, duration of ventricular repolarization; RR, intervals; QTcB, QT correction to HR.*

All participants were informed about the aim of the research, the possibility of refusal of the participation and provided written informed consent. The study was approved by the Local Bioethical Committee (Decision No 3/2018) and conducted in accordance with the Declaration of Helsinki of the World Medical Association.

### Study Protocol

All measurements were made in a pool environment with a water temperature of 27°C wearing a 5 mm-thick for STA and 1 mm-thick for dynamic apnea diving suits. Each diver performed two tests organized according to the (AIDA, 2021) competition protocol. The first immersion consisted in a STA, while the second consisted in a dynamic apnea without fins (DNF). The time between the two immersion protocols was 10 min. The BHT was recorded from the moment the face was immersed and finished when a diver emerged from the water. During the study protocol, the participants were controlled with a safety diver.

**FIGURE 1:**
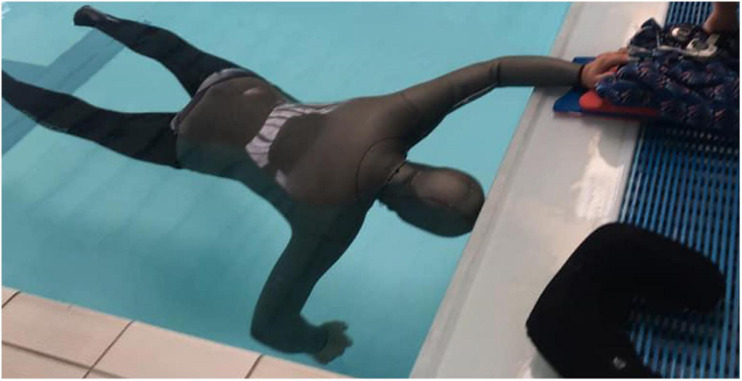
Static breath-hold test in water immersion (STA).

Before the STA test, all subjects performed a warm-up in which they repeated diving with 30, 50, and 60% of their maximum breath-hold-time obtained in the last 6 months. Then, the subjects started the STA test by performing hyperventilation of individual duration, inhaled to the maximum and dived into the swimming pool in a horizontal position (Figure 1) for a total immersion time of 70% of the individual maximum breath-hold time (BHT). After the STA test, the freedivers changed their diving suit and proceeded to the DNF test, in which the combination of apnea with exercise (swimming distance) was additionally assessed.

### Measurements and Biochemical Analyses

An assessment of the heart rate (HR) and blood oxygen saturation (SpO_2_) was continuously monitored during STA and before and immediately after dynamic apnea trials. To compare the results of STA, the values measured at 30, 50, and 70% of the subject’s BHT were included.

At the beginning of the study (rest) and the end of the breath-hold test (post DNF test), all subjects had venous blood drawn for the determination of HSPs (HSP 70, HSP 27, and HSP 90), NO, total oxidant status and total oxidant capacity (TOS/TOC), and TAS.

Total oxidant status and total oxidant capacity showing the total lipid peroxide concentration directly related to the level of oxygen radicals could be a good representative of the level of oxidative stress in biological fluids (Sadowska-Krępa et al., 2021). The reference ranges for TOS/TOC <200, 200–350, and >350 μmol/l correspond to low, moderate, and high oxidative stress, respectively.

Blood samples from the antecubital vein were collected to separator tubes and, after 30 min, centrifuged for 20 min at 1,000× *g*. Obtained serum was kept frozen at −80°C until analyzed.

The serum HSPs levels were measured by enzyme-linked immunosorbent assay ELISA Kit (ELISA Cloud-Clone Corp, Germany). The intra-assay and inter-assay coefficients of variation values were <12.0% and the test sensitivity was 1.33 ng/mL for serum HSP70 concentrations. Intra- and inter-assay coefficients of variation (CV) for HSP27 were <12.0% and the test sensitivity was 0.31 ng/mL, and for serum levels of HSP90, the CV values were <12% and the test sensitivity was 1.22 ng/mL.

The total NO and nitrate/nitrite (xNO) parameters were measured by Assay Kit (R&D System, BIOTechne Brand, North America). Intra-assay precision was 2.5%, and inter-assay precision was 4.6%. Total NO, nitrite, and nitrate levels in various samples were measured with a sensitivity of 0.78 μmol/L, and the assay range was 3.1–200 μmol/L.

Total antioxidant status was measured by enzyme immunoassay (Randox UK, NX 2332), and total oxidant status (TOS/TOC) was determined using the PerOx diagnostic kit and enzyme immunoassay (TOS/TOC) ELISA Kit (Germany, REF 5100) with a sensitivity of 7 μmol/L and CV <6.63%. Plasma samples for TAS and TOS were stored at −20°C until biochemical analyses were performed.

Biochemical analyses were performed in our certified laboratory, meeting the requirements of PN EN-ISO 9001:2009 (certificate No. 129/2015).

### Statistical Analyses

Descriptive statistics were calculated, and the results were presented as means and standard deviations (mean ± SD). All analyses were performed using the Statistica v. 10 statistical software package (StatSoft, Tulsa, OK, United States). The data were analyzed by one-way ANOVA followed by the Bonferroni test and the *U* Mann-Whitney test when appropriate. The statistical analysis includes a one-way ANOVA (rest vs. post-test), and Spearman correlation coefficients were analyzed to determine the inter-variable relationships. Statistical significance was set at *p* < 0.05.

## Results

The physical performance of the subjects, expressed as maximal oxygen consumption (VO_2max_), was 41.8 ± 4.7 ml/kg/min. There were no significant differences between the plethysmography variables (TLC, RV, IC, and DLCO) and ECG variables of the studied group compared to references norms for this age group. A tendency to higher inspiratory capacity, expiratory reserve volume, and DLCO compared to the predicted values was observed (Table 2). A comparison between baseline and simulation STA test showed a trend toward slower heart rate rhythm (HR) and a longer time for ventricular depolarization (QRS) compared to rest. At the basis of ECG analyses no cardiac function abnormalities in the participants were found.

**FIGURE 2:**
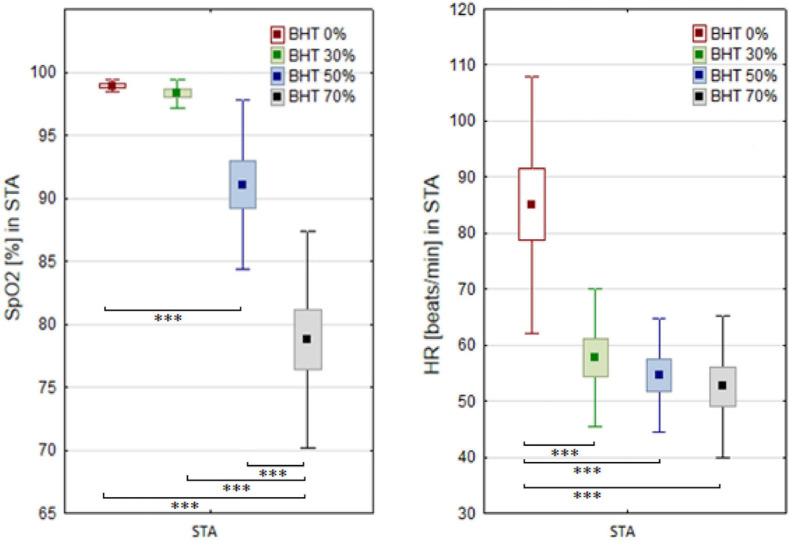
Blood oxygen saturation (SpO_2_) and heart rate (HR) changes before (BHT 0%) and at 30, 50, and 70% of breath holding time (BHT) during static apnea (STA). Significant differences ^∗∗∗^*p* < 0.001.

A significant effect of breath-hold and immersion on SpO_2_ and HR was demonstrated in the STA test (Figure 2). Significant differences in the SpO_2_ levels were observed between the values measured at 50 and 70% BHT compared to the bassline levels (*p* < 0.001 and *p* < 0.001, respectively). In the STA test, significant differences in the HR were demonstrated throughout the whole apnea time (*p* < 0.001).

Comparison of breath-holding time between the STA and DNF tests clearly indicated the effect of exercise on decrease apnea tolerance (*F* = 85.2; *p* < 0.001). There was a significant effect of apnea and exercise (DNF) on post-test HR (*F* = 35.6; *p* < 0.001). Higher BHT during the STA test corresponds to a significant decrease of HR but not SpO_2_ levels.

A negative correlation was observed between 70% of BHT and SpO_2_ (*r* = −0.74; *p* < 0.05). Significant correlation was observed between SpO_2_ measured during 70%BHT and TLC *r* = −0.59; *p* < 0.05) as well as SpO_2_ and DLCO (*r* = −0.61; *p* < 0.05).

**FIGURE 3:**
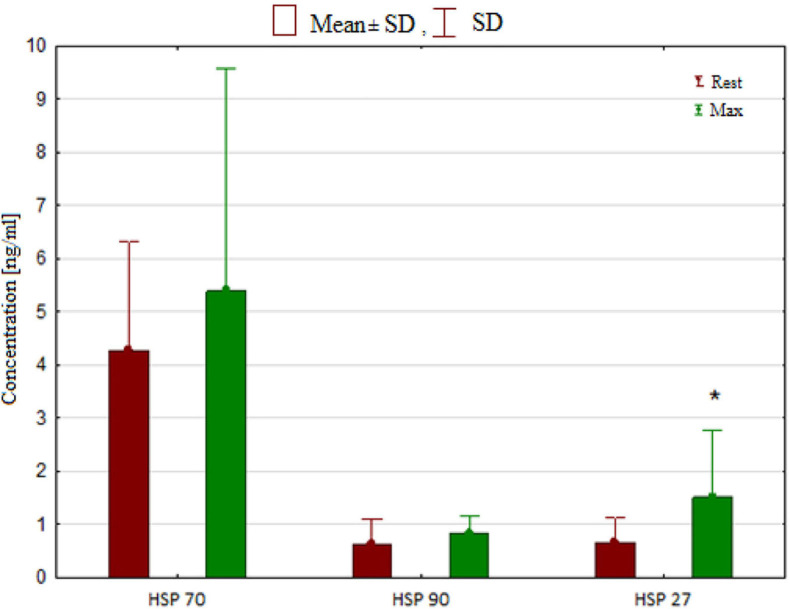
Concentration of heat shock proteins (HSP) in rest and after breath hold diving. Significant differences *p < 0.05.

The effects of breath-hold diving on serum HSPs and NO concentrations in freedivers were compared to baseline levels (Table 4). Analysis of variance revealed a significant effect of breath-hold diving protocol on serum HSP27 concentration (6.3; *p* = 0.03). ANOVA showed a non-significant effect of the intervention (rest vs. post-test) on serum total NO level (*p* > 0.05). ANOVA revealed a significant effect of the intervention on (*F* = 18.2; *p* < 0.001) on serum PerOx levels. A significantly higher post-test PerOx concentration was observed compared to the rest values (*p* < 0.001). There were no significant changes in the HSP70, HSP90 (Figure 3), and TAS under the influence of the static and dynamic breath-hold protocol (Table 4).

**TABLE 4:** Results of selected biochemical indices before and after breath hold diving.

**Variables**	***n* = 13**				***p***	***F***	**Post-hoc**
	**Rest**		**Post test**				
	***X***	**SD**	***X***	**SD**			
HSP 70 (ng/ml)	4.26	1.97	5.39	4.03	0.20	1.88	0.20
HSP 90 (ng/ml)	0.62	0.45	0.82	0.34	0.18	2.17	0.18
HSP 27 (ng/ml)	0.66	0.45	1.50	1.20	0.03	6.26	0.03
NO (μmol/L)	45.3	21.5	41.2	17.9	0.22	1.68	0.14
PerOx (μmol/L)	226.79	118.12	308.99	168.73	0.002	16.14	0.00
TAS (mmol/L)	1.47	0.11	1.50	0.14	0.15	2.07	0.15

*HSP, heat shock protein; NO, total nitric oxide; PerOx, lipid peroxidation products; TAS, total antioxidant status.*

A significant positive correlation between post-test HSP70 and TAS (*r* = 0.63; *p* < 0.05) was observed. An inverse correlation between post-test TAS and SpO_2_ (*r* = −0.70; *p* < 0.01) was also revealed. Significant positive correlations were detected between post-test NO and HSP70 (*r* = 0.88; *p* < 0.001); NO post-test and HSP90 (*r* = 0.59; *p* < 0.05); and NO post-test and HSP 27 (*r* = 0.66; *p* < 0.05).

## Discussion

The present study investigated whether breath-holding will induce adaptive cardiovascular mechanisms during the combined contribution of two factors hypoxic exposure STA and hypoxia and exercise training during dynamic apnea in elite freedivers. It also aimed to determine if this effect, if present, is accompanied by changes in serum levels of NO, HSPs (HSP27, HSP70, and HSP90), and blood antioxidant status.

Our results demonstrate an increase RR, the interval time (PR) and ventricular repolarization time (QT/QTc) and a decrease sinus rhythm the after breath-hold maneuver during STA test compared to the baseline values in freedivers. Significant changes in the QT interval depend on the frequency of heart depolarizations. This study may indicate a significant relationship between bradycardia and QT prolongation in response to STA. Breath-hold diving did not adversely affect the heart function of elite divers who participated in this study, and the analyzed variables fell within the reference values (Charbit et al., 2006; Mason et al., 2007). Freedivers had normal electrocardiographic parameters both at rest and after the STA test. The results of this study confirm the latest reports in the study of Kafes et al. (2020), who observe the lower risk of cardiac dysfunction by monitoring the ECG and hemodynamic parameters after maximum breath-hold of 24 athletes participating in the freediving competition. Similarly with our study, in divers with an average BHT of 113 s and low SpO_2_ value (88%), normal heart function was demonstrated (Laurino et al., 2012).

It has been evidenced that maximal apnea time in divers was accompanied by marked oxygen desaturation (Stewart et al., 2005; Willie et al., 2015). At the end of apnea, divers showed a >5-fold greater muscle sympathetic nerve activity increase with increased vascular resistance. The rise in muscle sympathetic nerve activity correlated with oxygen desaturation and with the increase in mean arterial pressure (Lemaitre et al., 2005; Heusser et al., 2009). Contrary to these results, cardiac arrhythmias by monitoring the electrocardiogram (ECG) were also found in 12 out of 16 recreational divers during voluntary immersed breath holds. It has been suggested that the occurrence of cardiac arrhythmias was significantly associated with BHT and was associated with individual factors, such as the tolerable SpO_2_ decrease (Hansel et al., 2009).

In our study, the average duration of breath-hold in elite athletes was 269 ± 62 s in the STA test and was significantly longer compared to results presented in previous studies (Hansel et al., 2009; Laurino et al., 2012). There was a significant reduction in HR and SpO_2_ depending on BHT, and significantly lower values were observed during the STA test compared to the dynamic test (DNF). On this basis, we can conclude that the training used in the studied group of freedivers increased apnea tolerance. However, a limitation of our study is the lack of the possibility of referring to the values of cardiovascular indices in the respondents to the values before their training period. Importantly, apnea time was negatively correlated with SpO_2_ at the level of 70% of maximum apnea, and lower SpO_2_ was associated with higher lung capacity (TLC) and diffusing capacity for carbon monoxide (DLCO). These results might indicate an increase in lung volume and function as an important factor in adapting the mechanism to freediving (Overgaard et al., 2006). Several other adaptive mechanisms cannot be ruled out that protect against hypercapnia and hypoxemia developing during apnea (Lindholm and Lundgren, 2008; Bain et al., 2018; Taboni et al., 2019; Elia et al., 2021).

From the literature data, it may be suggested that the slowing of the HR was the result of the activation of cardiac vagal fibers following the stimulation of peripheral chemoreceptors by hypoxemia (Lemaitre et al., 2005, 2008; Wierzba and Ropiak, 2011; Willie et al., 2015). Moreover, stimulation of parasympathetic fibers leads to increased bradycardia resulting from immersing the face in the water (Lemaitre et al., 2015). The phasic HR responses throughout a dry, static breath-holding in elite divers (Perini et al., 2008) and three distinct phases (i.e., an initial reduction-phase I, plateau-phase II, and further reduction-phase III) have been observed. The results of the presented study do not clearly confirm the phase HR changes. It should be emphasized that the bradycardia observed was 30% of BHT might be explained by an increase in the sensitivity of diving reflex receptors due to long-term apnea training.

Molecular mechanisms protecting against tissues and myocardium ischemia and hypoxia, although well known in clinical studies, are relatively rarely assessed in research data of athletes who practice diving (Marongiu et al., 2015; Zelenkova and Chomahidze, 2016). The protective mechanisms that have been confirmed in previous studies include: expunction of brain vessels due to the increase in carbon dioxide levels, increased dissociation of oxygen from hemoglobin, and the use of oxygen reserves (Tocco et al., 2012; Cross et al., 2013).

Previously, a significant role for NO and the HSPs in adapting the vascular system (Andreadou et al., 2015) and important cardioprotective factors after repeated breath-holds has also been considered (Joulia et al., 2009; Marlinge et al., 2019; Cialoni et al., 2021b). The most important results of this study include the increased release of HSPs (HSP 27) and the tendency of a higher concentration of HSP 70 and HSP 90 after the end of breath-hold trials compared to the resting values. Post static and dynamic apnea TOS was significantly greater in response to breath-holding maneuvers. We also found significant correlations between the post-test NO and HSP70, NO and HSP 90, and NO and HSP 27 levels. Serum HSP 70 concentration increased in response to apnea correlated with TAS. The observed increase of TAS was evident at the lowest SpO_2_. In our study, the TOS/TOC measured in response to static and dynamic apnea pointed to moderate oxidative stress. The results indicate that changes in oxygen availability (SpO_2_) during the breath-hold dive were associated with a significant increase in the total oxidant status (TOS/TOC) in the blood. Interestingly, changes in NO serum concentrations after performing breath-hold maneuvers may have no significant impact on the activation of protective vasoconstrictive mechanisms. However, the beneficial effects may be confirmed by the positive correlation between NO and HSPs levels.

Nitric oxide synthase (iNOS) and cyclooxygenase (COX-2) have been previously reported as important factors responsible for exercise-induced cardioprotection mechanisms (Bolli, 2007; Powers et al., 2014). Theunissen et al. (2013a, b) reported that during breath-hold diving, an increase in iNOS activity and superoxide anions levels had been observed as a result of transient hyperoxia followed by hypoxia and CO_2_ accumulation. Therefore, NO has been suggested as an important element in initiating cardioprotective signals. However, its excessive accumulation during ischemia might contribute to the formation of nitrites and, consequently, reperfusion damage by nitrogen stress (Andreadou et al., 2015). The induction of ROS production and nitrate concentration in response to changes in molecular oxygen pressures might affect endothelial function (Mrakic-Sposta et al., 2019). Similar to the presented study, there was also an increase in the TOS levels but significantly lower antioxidant capacity after diving was observed. Joulia et al. (2003) suggested that the presence of oxidative stress after breath-hold sessions, as evidenced by an increase in the concentration of reactive substances in thiobarbituric acid. Contrary, no changes in the level of secondary lipid peroxidation products and the decrease of superoxide dismutase activity have also been observed (Mila-Kierzenkowski et al., 2015). These authors suggested that inhibition of free-radical processes occurs and/or the products of lipid peroxidation are quickly removed due to adaptation, which protects the elite divers against damages on the cellular level.

Our findings support the hypothesis that physiological and molecular processes are responsible for increasing tolerance in hypoxic conditions in breath-hold divers during a combination of static and dynamic apnea. It has been suggested that exercise training increases the concentrations of basal levels of HSPs (Lin et al., 2001; Peake et al., 2015). The protective effect of HSPs has been documented in experimental animal studies in which exercise-induced HSPs reduced the risk of cardiovascular diseases (Demirel et al., 2003; Senf et al., 2008). An increase in HSPs is believed to protect the heart from ischemia-reperfusion damage by increasing its antioxidant capacity. The significant role of HSPs for mitochondria by protecting cells against apoptosis has also been suggested (Dimauro et al., 2016). In the present study, an increase in HSP 27 concentration after a few minutes of breath-hold during static and dynamic apnea indicates a contribution from this protein to a protective mechanism against hypoxia (Arya et al., 2007). Importantly, the obtained positive relationship between HSP 70 and TAS might indicate an important role of this protein in protecting cells against oxidative stress (Dimauro et al., 2016). It has been documented that exercise training facilitates the expression of HSPs in the heart (Demirel et al., 2003), increasing their cardio protective effects (Powers et al., 2014). In these studies, there was a trend toward higher concentrations of HSP 70 and HSP 90 depending on antioxidant defense (TAS) and a significant effect of the TOS in the blood, was also observed. Lipid peroxidation, the oxidation process of polyunsaturated fatty acids leading to the formation of peroxides of these compounds, is the best-known oxidation process initiated by free radicals. The levels of lipoperoxidation products referred to as the oxidative potential indicate the activity of ROS in the examined tissues (Finaud et al., 2006; Sureda et al., 2015).

We detected an increase in lipid peroxidation products after breath-hold and exercise, confirming the higher activity of the free radical process under the influence of the repeated apnea maneuvers. Surprisingly, we did not observe a significant increase in the antioxidant potential based on the TAS after breath-hold compared to resting values. It can be presumed that the state of antioxidant defense is not responsible for protecting cells against ROS in the studied group of divers. However, it cannot be ruled out that the determination of pro-oxidative-anti oxidative status in the minutes following the long-term recovery would indicate that such a defense was induced.

## Conclusion

Static and dynamic breath-holding had a significant effect on oxidative stress, as evidenced by an increase in the total oxidant status and total oxidant capacity. An increase in serum heat shock protein levels in response to higher nitric oxide levels could explain its important role in improving cardiovascular adaptation to hypoxia in elite freedivers.

Further studies are necessary to explain the exact mechanisms of breath holds training of cardiovascular adaptation responsible for maintaining adequate oxygen supply in elite divers.

## Data Availability Statement

The raw data supporting the conclusion of this article will be made available by the authors, without undue reservation.

## Ethics Statement

The studies involving human participants were reviewed and approved by Bioethical Committee of Academy of Physical Education (Decision No 3/2018). The patients/participants provided their written informed consent to participate in this study.

## Author Contributions

MS-T, AŻ, DJ, and PS contributed to conception and design of the study. MS-T, SK-N, RM, and DZ organized the database. RM performed the statistical analysis. MS-T and AŻ wrote the first draft of the manuscript. AŻ and PS wrote sections of the manuscript. All authors contributed to manuscript revision, read, and approved the submitted version.

## Conflict of Interest

The authors declare that the research was conducted in the absence of any commercial or financial relationships that could be construed as a potential conflict of interest.

## Publisher’s Note

All claims expressed in this article are solely those of the authors and do not necessarily represent those of their affiliated organizations, or those of the publisher, the editors and the reviewers. Any product that may be evaluated in this article, or claim that may be made by its manufacturer, is not guaranteed or endorsed by the publisher.
